# The native ant, *Tapinoma melanocephalum*, improves the survival of an invasive mealybug, *Phenacoccus solenopsis*, by defending it from parasitoids

**DOI:** 10.1038/srep15691

**Published:** 2015-10-27

**Authors:** Dong-Dong Feng, J.P. Michaud, Pan Li, Zhong-Shi Zhou, Zai-Fu Xu

**Affiliations:** 1 Department of Entomology, College of Nature Resources and Environment, South China Agricultural University, Guangzhou, China; 2 Department of Entomology, Agricultural Research Center-Hays, Kansas State University, Hays, Kansas, USA; 3 State Key Laboratory for Biology of Plant Diseases and Insect Pests, Institute of Plant Protection, Chinese Academy of Agricultural Sciences, Beijing, China

## Abstract

Mutualistic ants can protect their partners from natural enemies in nature. *Aenasius bambawalei* is an important parasitoid of the the invasive mealybug *Phenacoccus solenopsis*. We hypothesized that mutualism between native ants and mealybugs would favor survival of mealybugs. To test this, we examined effects of tending by the native mutualistic ant *Tapinoma melanocephalum* on growth of *P. solenopsis* colonies on Chinese hibiscus, *Hibiscus rosa-sinensis*, in a field setting. Ant workers with access to honeydew of mealybugs lived much longer than those provisioned only with water in the laboratory, and number of ant workers foraging increased significantly with growth of mealybug colonies in the field. In later observations, there were significant differences in densities of mealybugs between ant-tended and -excluded treatments. Survival rate of mealybugs experiencing parasitoid attack was significantly higher on ant-tended plants than on ant-excluded plants. When the parasitoid was excluded, there was no difference in survival rate of mealybugs between ant-tended and -excluded plants. In most cases, ants directly attacked the parasitoid, causing the parasitoid to take evasive action. We conclude that native ants such as *T. melanocephalum* have the potential to facilitate invasion and spread of *P. solenopsis* in China by providing them with protection from parasitoids.

Mutualistic interactions are ubiquitous phenomena occurring between many classes of organisms^1^,^2^,^3^,^4^, and quite commonly between ants and hemipterans^5^,^6^,^7^,^8^,^9^,^10^,^11^,^12^,^13^,^14^,^15^,^16^. Mutualisms between ants and hemipterans are among the most conspicuous and well-studied mutualistic interactions that occur among insects^5^,^6^,^9^,^17^,^18^. Hemipterans provide honeydew, a significant source of carbohydrates for the ants, while the ants protect the hemipterans against parasitoids and predators^9^,^18^. Consequently, the survival and persistence of hemipteran populations can be strongly enhanced by the presence of mutualistic ants^5^,^11^,^13^,^19^.

The solenopsis mealybug, *Phenacoccus solenopsis* Tinsley (Hemiptera: Pseudococcidae), is a species native to North America^20^ that has become a cosmopolitan pest of cotton^21^,^22^,^23^,^24^. This invasive species spread to Asia in 2005 and caused serious damage to cotton in Pakistan and India^23^,^25^. It was first discovered in Guangzhou City, Guangdong Province, China on hibiscus, *Hibiscus rosa-sinensis* L., and rapidly spread to infest many southern provinces^26^. Yield loss estimates due to *P. solenopsis* infestations in cotton were 1.4 million tons in China in 2008/2009^27^. Several parasitoid species have shown good potential for controling the pest^28^,^29^,^30^,^31^,^32^,^33^. One of the most dominant species is *Aenasius bambawalei* Hayat (Hymenoptera: Encyrtidae), a solitary parasitoid of *P. solenopsis*, which has shown promise suppressing populations of the mealybug in India, Pakistan and China^32^,^34^,^35^,^36^,^37^,^38^,^39^.

Since *P. solenopsis* secretes honeydew, it often engages in mutualistic interactions with ants, e.g., the red imported fire ant, *Solenopsis invicta* Buren^14^. Another study suggested that *S. invicta* utilized shelters constructed by the cotton leaf roller, *Sylepta derogata* F. (Lepid.: Pyralidae), to help shield *P. solenopsis* from its natural enemies^40^. However, these studies did not address whether this invasive ant provided direct protection of *P. solenopsis* against parasitoids or predators^14^,^40^.

Native ant species are abundant in the areas of China invaded by *P. solenopsis* and the ghost ant, *Tapinoma melanocephalum* (F.), is one of the most dominant. This species can be observed tending *P. solenopsis* and sometimes transporting individuals from lower to upper leaves on *H. rosa-sinensis* plants^41^, but we have not observed any predation of the mealybugs. To date, native ants retain a broader distribution in China than the red invasive fire ant, *Solenopsis invicta* Buren, so *P. solenopsis* may have many opportunities to establish mutualisms with native ants in the process of range expansion. Here, we address the question of whether mutualisms can evolve between native ants and *P. solenopsis*, and whether their aggressive behavior towards parasitoids can improve the survival of invasive mealybug colonies. We hypothesized that *P. solenopsis* colonies able to establish mutualistic relationships with native ants would grow larger and suffer less parasitism than colonies prevented from doing so. To test this hypothesis, a series of experiments were conducted to monitor the growth and survival of *P. solenopsis* colonies with and without the benefits of tending by *T. melanocephalum* workers. The results of these experiments may help us anticipate the potential role of native mutualistic ants in facilitating the invasion and range expansion of exotic honeydew-producing hemipterans.

## Results

### Mutualism in the field

**Figure 1:**
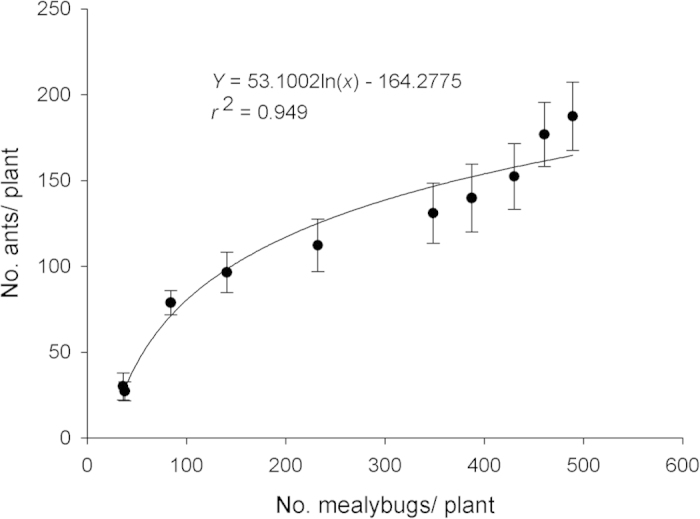
Relationship between mean numbers of ants foraging per plant and mean numbers of *P. solenopsis* as described by non-linear regression.

**Figure 2:**
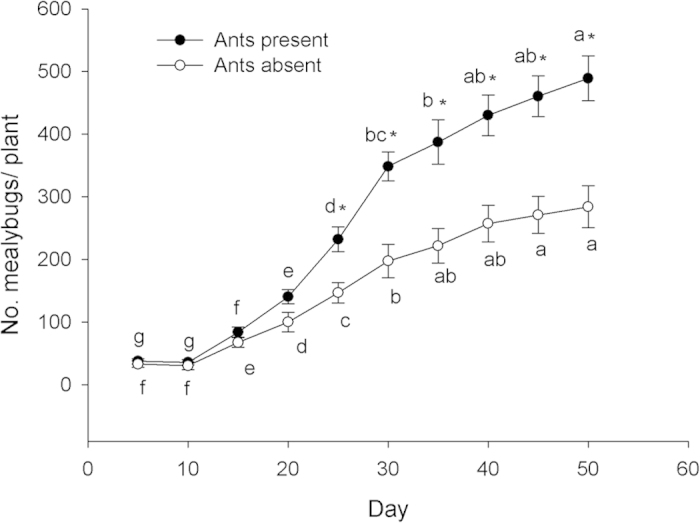
Mean number (±SE) of *P. solenopsis* per plant in ant-tended plots and ant-excluded plots. Values bearing the same letters were not significantly different from other dates within treatments (ANOVA for repeated measures, LSD, α = 0.05). Asterisks indicate significant differences between treatments within dates (ANOVA, α = 0.05).

There was a significant positive correlation between the numbers of ant workers foraging and numbers of *P. solenopsis* over time (*F* = 147.36; *df* = 1,8; *P* < 0.0001) with variation in one variable explaining almost 95% of variation in the other (Fig. 1). Densities of *P. solenopsis* differed significantly between ant-tended and ant-excluded treatments (*F* = 126.45; df = 1,4; *P* = 0.0004) and among sampling dates (*F* = 161.79; df = 9,72; *P* < 0.0001) and there was a significant treatment*sampling date interaction (*F* = 12.62; *df* = 9,72; *P* < 0.0001). Differences became significant on the fifth sampling date and remained different thereafter (Fig. 2).

### Longevity of *T. melanocephalum* workers fed *P. solenopsis* honeydew

**Figure 3:**
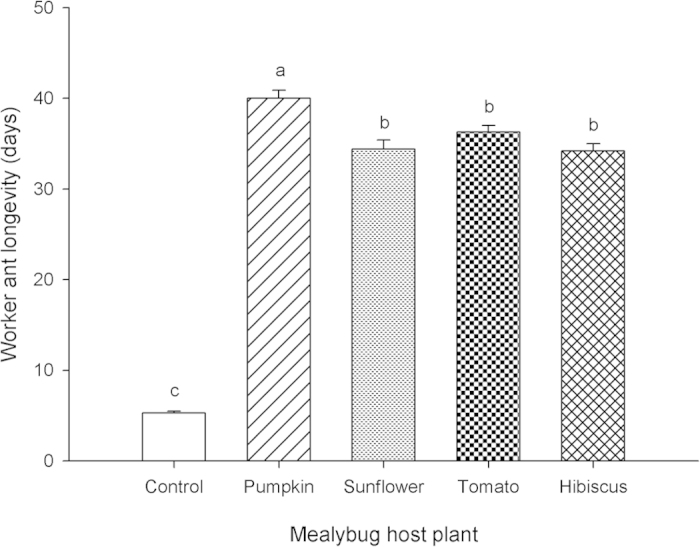
Mean (±SE) longevities of *T. melanocephalum* workers (n = 30 per treatment) fed *P. solenopsis* honeydew produced on four different host plants, versus purified water (control). Columns bearing the same letters were not significantly different from others (ANOVA followed by LSD, α = 0.05).

Mealybug honeydew produced on *C. moschata* yielded the greatest worker ant longevity, but access to all types of *P. solenopsis* honeydew prolonged worker ant longevity significantly relative to purified water (*F* = 328.18; *df* = 4,145; *P* < 0.0001; Fig. 3).

### Parasitism by *A. bambawalei* in the presence of ants

**Figure 4:**
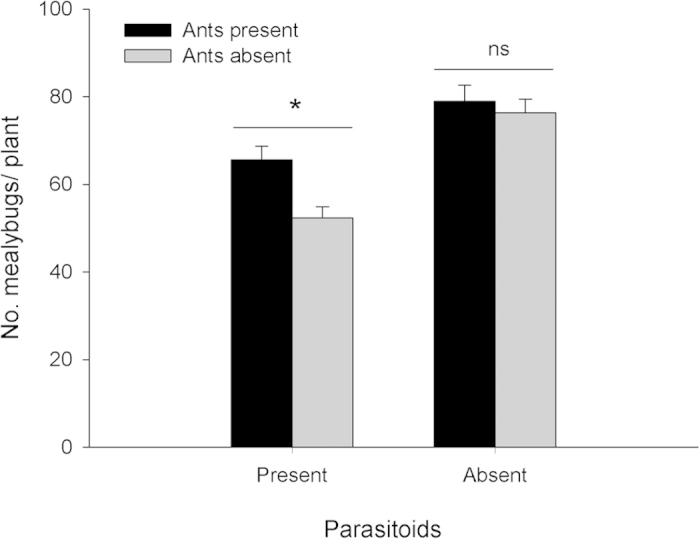
Mean (+SE) numbers of *P. solenopsis* per plant with and without ants and parasitoids present. Asterisk indicates a significant difference between columns with parasitoids present (ANOVA, α = 0.05).

When *A. bambawalei* was present, the presence of ants resulted in larger numbers of *P. solenopsis* at end of experiment than in their absence (*F* = 10.56, *df* = 1,8, *P* = 0.0117, Fig. 4), but there was no effect of ant tending when parasitoids were not present (*F* = 0.30, *df* = 1,8, *P* = 0.5986). Percentage parasitism of *P. solenopsis* by *A. bambawalei* was lower on plants with ants (mean ± SE = 34.5 ± 3.2%) than on plants without them (mean ± SE = 47.7 ± 2.5%; *F* = 10.65, *df* = 1,8; *P* = 0.0115).

### Ant-parasitoid behavioral interactions

**Figure 5:**
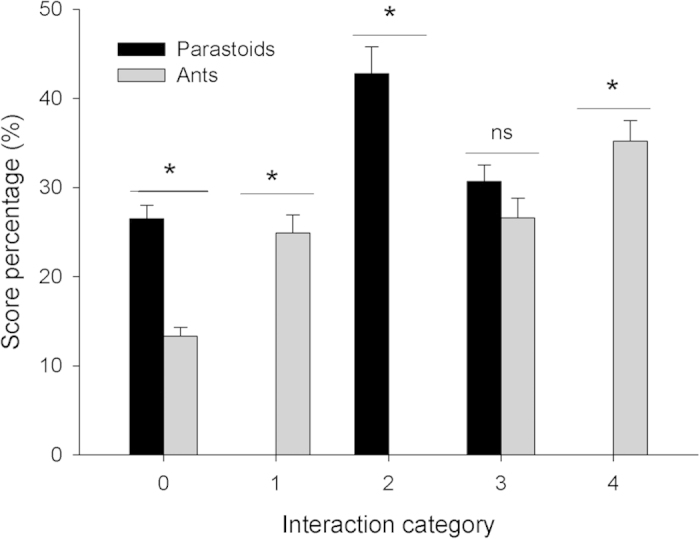
Percentage of time spent by the parasitoid *A. bambawalei* and the ant *T. melanocephalum* in four different categories of interaction (0 = ignore/mutual non-interference; 1 = touch, antennation or grooming; 2 = avoidance or evasive behavior following contact; 3 = dorsal flexion or other defensive reaction; 4 = fighting, prolonged aggression, sparring, charging, biting and pushing. Asterisks indicate significant differences between parasitoids and ants within an aggressive interaction category.

We observed higher levels of aggression by *T. melanocephalum* against *A. bambawalei* than vice versa. Ants attacked parsasitoids but parasitoids did not attack ants (interaction category 4: *χ*^*2*^ = 42.7184, *df* = 1, *P* < 0.0001, Fig. 5), and spent more time in avoidance or escape behaviors (interaction category 2: *χ*^*2*^ = 54.4529, *df* = 1, *P* < 0.0001) and in states of mutual non-interference (interaction category 0; *χ*^*2*^ = 5.4655, *df* = 1, *P* = 0.0.0194). Ants often contacted and antennated parasitoids, but not the reverse (interaction category 1; *χ*^*2*^ = 54.4529, *df* = 1, *P* < 0.0001). Both *T. melanocephalum* and *A. bambawalei* employed dorsal flexion and other defensive tactics to a similar extent (interaction category 3; *χ*^*2*^ = 0.4112, *df* = 1, *P* = 0.5214).

## Discussion

There are numerous species of native ants with extensive distribution throughout China, providing *P. solenopsis* with many opportunities to establish mutualisms that may enhance its invasion and spread. We chose a dominant native ant species, *T. melanocephalum*, to test whether mutualism with a native ant would improve survival of this invasive mealybug. The greater increase in population density of *P. solenopsis* in the ant-tended treatment than in the ant-excluded treatment demonstrates the benefit of ant tending for the mealybugs, and the increased longevity of workers in the laboratory feeding trial suggests a reciprocal benefit for the ants. Furthermore, the positive correlation between numbers of *P. solenopsis* and numbers of ants foraging indicates that *T. melanocephalum* workers recruit in direct proportion to the numbers of *P. solenopsis* present (Figure S1). Many previous studies have shown that hemipterans provide honeydew for ants that supports the growth of their colony^5^,^6^,^7^,^9^,^11^,^14^. For example, the red imported fire ant, *S. invicta*, can utilize honeydew produced by *P. solenopsis* to promote colony growth^14^.

In return for carbohydrates, ants often protect their mutualistic partners from natural enemies^2^,^5^,^11^,^17^,^18^. For example, the role of ants as guardians of aphids against predators and parasitoids is well documented^3^,^10^,^13^,^18^,^19^,^42^,^43^,^44^,^45^ and similar mutualisms have evolved between ants and other hemipterans^5^,^6^,^9^,^14^,^17^ and butterflies^2^,^46^,^47^,^48^. In the present study, the parasitoid *A. bambawalei* parasitized more *P. solenopsis* on ant-excluded plants than on ant-tended plants, such that the colonies of *P. solenopsis* grew significantly larger on the latter. When the parasitoid was excluded, there were no differences in numbers of *P. solenopsis* between ant-excluded and ant-tended plants, clear evidence that *T. melanocephalum* acts to protect *P. solenopsis* from *A. bambawalei*. These results are consistent with the findings of previous work in India^49^,^50^.

Differences in levels of interspecific aggression between the ant and the parasitoid were evident in the behavioral observations. A high level of interspecific aggression was directed toward *A. bambawalei* by *T. melanocephalum* that was not reciprocated; the parasitoid responded to ant encounters mostly with evasive movements and defensive postures. In summary, the present study provides several lines of evidence to indicate that native ants will likely reduce the effectiveness of the introduced parasitoid *A. bambawalei* and promote the successful invasion and spread of *P. solenopsis* in China.

## Methods

### Plants

To assess the benefits of honeydew consumption by ants, seeds of Chinese hibiscus, *Hibiscus rosa-sinensis* L., calabaza squash, *Cucurbita moschata* Duch. Ex Lam, tomato, *Solanum esculentum* L. and sunflower, *Helianthus annuus* L., were sown in plastic flowerpots (18 cm diam) filled with a loamy clay soil. Plants were grown in a greenhouse at 26–30 ^o^C, 65 ± 5% RH, and a 14:10 L:D photoperiod, watered once every four days, and fertilized (N:P:K = 13:7:15) twice a month. These plants were infested with mealybugs when they reached a height of about 20 cm. Plants of *H. rosa-sinensis* for cage experiments with ants were grown in the same manner, but were used in experiments when they reached a height of 50 cm.

### Insects

None of the study species are protected in China, so no specific permits were required for collections or field activities. All source material was collected from plants of *H. rosa-sinensis* on the campus of South China Agricultural University, Guangzhou, Guangdong Province, China. A colony of *P. solenopsis* was established on *H. rosa-sinensis* plants that had been grown in 18 cm diameter plastic flowerpots in a greenhouse. The potted plants were used in experiments when they were ca. 50 cm tall with 50–60 true leaves. Each plant was infested by transferring ca. 60 first instar mealybug nymphs directly to the leaves. Each plant was then isolated in a ventilated aluminum frame cage (60 cm × 60 cm × 60 cm). Four generations were reared to obtain sufficient insects for use in experiments. All mealybug colonies were reared in the laboratory at 27 ± 1 ^o^C and a relative humidity of 60–70%. Third instar nymphs were used in experiments.

Mealybug nymphs parasitized by *A. bambawalei* were collected from *H. rosa-sinensis* plants on the campus of South China Agricultural University and reared out in the laboratory. Parasioids emerging from mummified mealybugs were identified and raised for four generations using ca. 100 adult females to start each generation. All wasps used in experiments were newly emerged adults. All parasitoid colonies were reared in the laboratory under the same physical conditions as the mealybugs with a 10% honey solution provided on cotton balls as food for the parasitoid adults.

We found a total of 11 native ant species on the university campus, with *T. melanocephalum* the most abundant species^41^. Workers of *T. melanocephalum* were collected from *Hibiscus rosa-sinensis* plants on the campus and fed on a 10% honey solution. Six newly established colonies of *T. melanocephalum* were collected from the campus of South China Agricultural University, each including one queen and a collection of adult workers, eggs, larvae and pupae. Each ant colony was housed in a 1.5 L plastic box and provisioned with a 10% honey solution in tubes with cotton wicks.

### Mutualism in the field

Two plots of about 50 m^2^ (10 m × 5 m) were planted with Chinese hibiscus, *H. rosa-sinensis*, in July, 2012. Experiments were initiated when the plants reached a height of about 60 cm and continued until January, 2013. Two treatments were established as follows: (1) Five *H. rosa-sinensis* plants without ant infestation were selected and marked; fifty second instar mealybug nymphs were then placed on the leaves and the base of the main stem was then coated with paraffin to exclude ants. (2) Five *H. rosa-sinensis* plants without ant infestation were selected and marked; fifty second instar mealybug nymphs were then placed on the leaves of the chosen plants, but no paraffin was applied to the stems of these plants. The number of ants foraging and numbers of live *P. solenopsis* on each plant was recorded for a period of five minutes every five days until a total of 10 observations were obtained.

### Longevity of *T. melanocephalum* workers fed *P. solenopsis* honeydew

The fitness benefits of *P. solenopsis* honeydew consumption by *T. melanocephalum* were assessed by measuring ergate longevity when fed mealybug honeydew produced on four different host plants: *H. rosa-sinensis, C. moschata, S. esculentum* L. and *H. annuus*. A total of 150 adult female mealybugs were placed on leaves of each potted plant (ca. 20 cm ht) and a series of glass Petri dishes (9.0 cm diam) were then placed under the plants. Honeydew excreted by *P. solenopsis* was collected in the Petri dishes after 24 h and dissolved with purified water to form a 10% solution. The dissolved honeydew was provisioned to ant workers individually (n = 30 per treatment) on cotton balls (1.5 cm diam) in glass Petri dishes (5.5 cm diam) with purified water as a control, all refreshed daily until the ant died.

### Parasitism by *A. bambawalei* in the presence of ants

In order to assess the impact of ants on mealybug parasitism by *A. bambawalei* under controlled conditions, a cage experiment was conducted in a greenhouse at 27–34 ^o^C and 50–75% RH. Four different treatments (n = 5 replications per treatment) were established, each employing a single potted *H. rosa-sinensis* plant (ca. 50 cm ht) in a ventilated aluminum cage (as above) infested by placing 100 third instar mealybug nymphs on the youngest leaves. Treatments were as follows: 1) mealybugs only (control), 2) mealybugs + ants, 3) mealybugs + parasitoids, 4) mealybugs + ants + parasitoids. For treatments including ants, an artificial nest of *T. melanocephalum* comprised of one queen and ca. 800 workers were transferred to each cage 24 after infestation with mealybugs. A plastic hose was used to build a bridge between the ant nest and the bottom of the plant to allow foraging workers to access the plant^14^. For treatments including parasitoids, once ants had colonized the plant, eight pairs of *A. bambawalei* adults were released in the cage and left to forage for 24 h. After two weeks, the number of the mealybugs was counted on all plants in the experiment. In addition, the numbers of parasitized mealybug nymphs were recorded on plants with and without ants.

### Ant-parasitoid behavioral interactions

For each experimental replicate, a fresh *H. rosa-sinensis* leaf was placed in a glass petri dish (Φ 9.0 cm), and ten third instar mealybug nymphs were placed in the center of the leaf. An ant and a parasitoid were introduced into the dish simultaneously, and the behavior of *T. melanocephalum* toward *A. bambawalei* directly observed during a 10 min period of interaction. Interactions were scored for each insect as described in^51^: 0 = ignore/mutual non-interference; 1 = touch, antennation or grooming; 2 = avoidance or evasive behavior following contact; 3 = dorsal flexion or other defensive reaction; 4 = fighting, prolonged aggression, sparring, charging, biting and pushing. The experiment was replicated five times with ten pairs of insects in each replication for a total of 50 trials. The mean of all replications was used to tally a score for each interaction category for both ant and parasitoid.

### Statistical Analyses

Mutualism in the field: Logarithmic regression was used to describe the relationship between the numbers of ant workers foraging and numbers of *P. solenopsis* over time. A 2-way ANOVA for repeated measures was used to test for significant differences between treatments and determine the date on which ant-tended plants became significantly different from ant-excluded plants. Longevity of *T. melanocephalum* workers fed *P. solenopsis* honeydew: Data on the response variable ‘ant longevity’ were found to be normally distributed and were subjected to one-way ANOVA followed by Fisher’s LSD (α = 0.05) to separate means among independent variables (plant species). Parasitism by *A. bambawalei* in the presence of ants: A one-way ANOVA was used to analyze treatment effects (presence of ants and/or parasitoids) on the response variables ‘no. mealybugs’ and ‘percent parasitism’. Ant-parasitoid behavioral interactions: A Chi Square, Goodness of Fit test was used to test for differences between ants and parasitoids in proportion of time spent in different categories of aggressive behaviors. All statistical analyses were conducted using SPSS version 16.0 (SPSS Inc., Chicago, IL, USA).

## Additional Information

**How to cite this article**: Feng, D.-D. *et al.* The native ant, *Tapinoma melanocephalum*, improves the survival of an invasive mealybug, *Phenacoccus solenopsis*, by defending it from parasitoids. *Sci. Rep.* **5**, 15691; doi: 10.1038/srep15691 (2015).

## Supplementary Material

Supplementary Information
