# Supplementary material for: The native ant, Tapinoma melanocephalum, improves the survival of an invasive mealybug, Phenacoccus solenopsis, by defending it from parasitoids
Source: Sci Rep. 2015 Oct 27;5:15691. doi: 10.1038/srep15691 (PMC4621601; doi:10.1038/srep15691)
Supplement: Supplementary Information [file srep15691-s1.doc]

**The native** **ant, *Tapinoma melanocephalum*, improves the survival of an invasive mealybug, *Phenacoccus solenopsis*, by defending it from parasitoids**

**Dong-Dong Feng^1^,** **J.P. Michaud^2^, Pan Li^1^, Zhong-Shi Zhou^3,*^and Zai-Fu Xu^1,*^**

^1^Department of Entomology, College of Nature Resources and Environment, South China Agricultural University, Guangzhou, China

^2^Department of Entomology, Agricultural Research Center-Hays, Kansas State University, Hays, Kansas, USA

^3^State Key Laboratory for Biology of Plant Diseases and Insect Pests, Institute of Plant Protection, Chinese Academy of Agricultural Sciences, Beijing, China

***Running head:*** Ants defend invasive mealybugs from parasitoids

**Dong-Dong Feng:** Wushan Street, Tianhe District, Guangzhou, 510642 China; dongdongfeng@foxmail.com.

**Pan Li:** Wushan Street, Tianhe District, Guangzhou, 510642 China; [pan1006pan@qq.com](mailto:pan1006pan@qq.com).

**J.P. Michaud:** 1232 240th Ave., Hays, KS, 67601 USA; jpmi@ksu.edu.

**Zhong-Shi Zhou**: # 2, West Road, Yuan-Ming-Yuan, Beijing, 100193 China; [zhongshizhou@yahoo.com](mailto:zhongshizhou@yahoo.com).

**Zai-Fu Xu**: Wushan Street, Tianhe District, Guangzhou, 510642 China; [xuzaifu@scau.edu.cn](mailto:xuzaifu@scau.edu.cn).

******Corresponding author:*** Prof. Zhong-Shi Zhou, Tel: +86 10-62810159 and Prof. Zai-Fu Xu, Tel: +86 20-85282321.


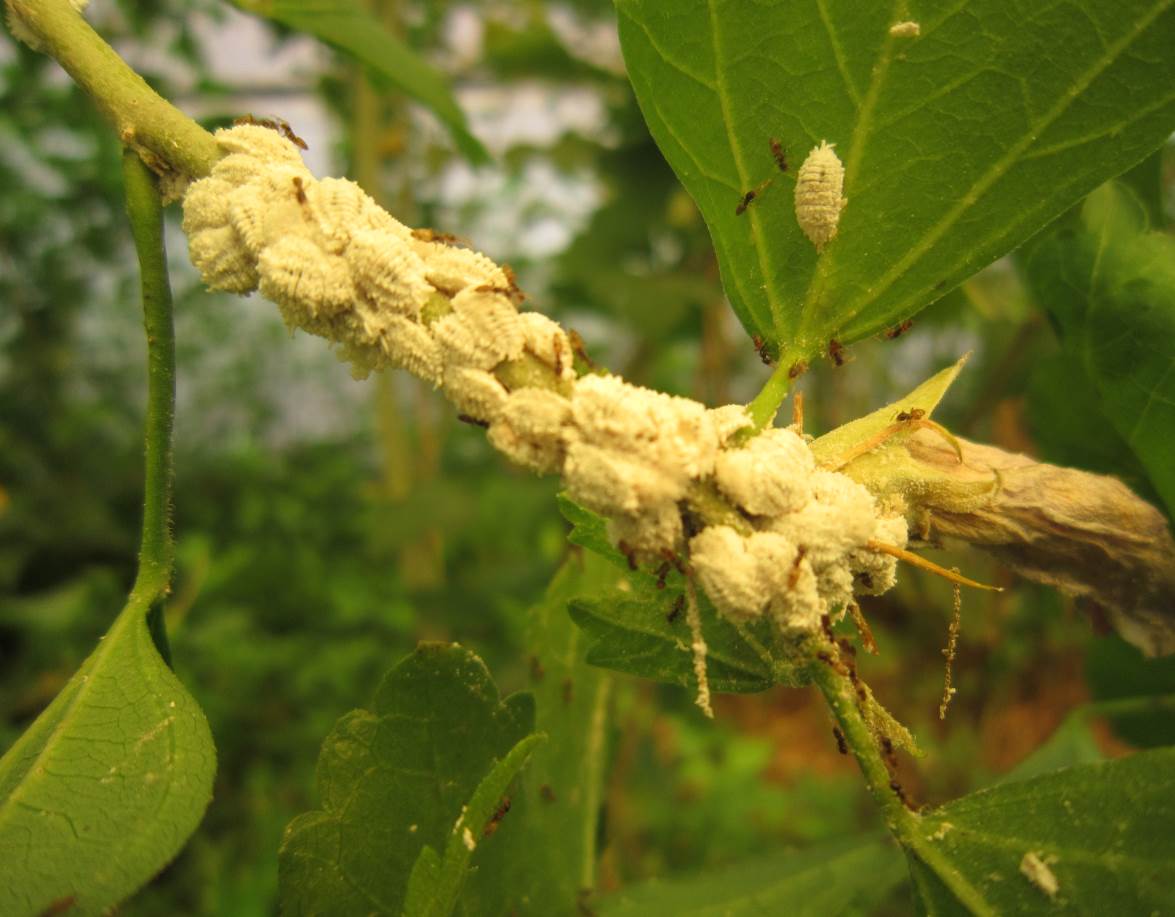


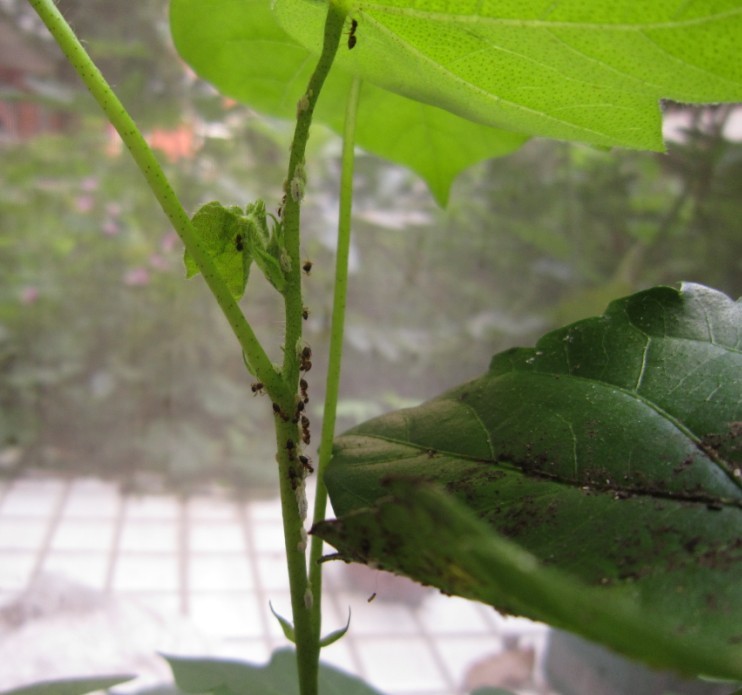


**Figure S1** Workers of *Tapinoma melanocephalum* tending a colony of *Phenacoccus solenopsis* on a hibiscus plant. Photograph taken by Dong-Dong Feng.
